# Role of factor H-related protein 3 in *Pseudomonas aeruginosa* bloodstream infections

**DOI:** 10.3389/fimmu.2024.1449003

**Published:** 2024-09-04

**Authors:** Alex González-Alsina, Héctor Martín-Merinero, Margalida Mateu-Borrás, María Verd, Antonio Doménech-Sánchez, Joanna B. Goldberg, Santiago Rodríguez de Córdoba, Sebastián Albertí

**Affiliations:** ^1^ Instituto Universitario de Investigación en Ciencias de la Salud (IUNICS), Universidad de las Islas Baleares and Instituto de Investigación Sanitaria de les Illes Balears (IDISBA), Palma de Mallorca, Spain; ^2^ Center for Biological Research-Margarita Salas and Centro de Investigación Biomédica En Red (CIBER) de Enfermedades Raras, Madrid, Spain; ^3^ Department of Pediatrics, Emory-Children’s Cystic Fibrosis Center, Division of Pulmonary, Asthma, Cystic Fibrosis, and Sleep, Emory University School of Medicine, Atlanta, GA, United States

**Keywords:** FHR-3, factor H, complement system, *P. aeruginosa*, bloodstream infection

## Abstract

Pseudomonas aeruginosa is a leading cause of nosocomial bloodstream infections. The outcome of these infections depends on the virulence of the microorganism as well as host-related conditions and factors. The complement system plays a crucial role in defense against bloodstream infections. *P. aeruginosa* counteracts complement attack by recruiting Factor H (FH) that inhibits complement amplification on the bacterial surface. Factor H-related proteins (FHRs) are a group of plasma proteins evolutionarily related to FH that have been postulated to interfere this bacterial evasion mechanism. In this study, we demonstrate that FHR-3 competes with purified FH for binding to *P. aeruginosa* and identify EF-Tu as a common bacterial target for both complement regulator factors. Importantly, elevated levels of FHR-3 in human serum promote complement activation, leading to increased opsonization and killing of *P. aeruginosa*. Conversely, physiological concentrations of FHR-3 have no significant effect. Our findings suggest that FHR-3 may serve as a protective host factor against *P. aeruginosa* infections.

## Introduction

*Pseudomonas aeruginosa* is one of the most common Gram-negative organisms causing nosocomial bloodstream infections with a high-rate mortality ranging from 30% to about 50% (1–4). The reasons for the poor outcome of these infections are multifactorial and include the frequent emergence of multidrug-resistant strains, the intrinsic virulence of the pathogen, and host related-factors (5, 6). A remarkable feature of *P. aeruginosa* bloodstream infections is their rapid progression. Many deaths occur shortly after infection (3, 4), suggesting that an altered host’s early innate immune system response predisposes to this type of infection.

The complement system is a crucial early innate immune effector and the main defensive mechanism against *P. aeruginosa* in blood (7). Complement is activated by three different pathways: classical, lectin and alternative pathway. The result of the activation of any of the three pathways is the formation of C3 convertases which cleave C3 to generate C3b molecules that are deposited on the bacterial surface. Surface-bound C3b organizes additional C3 convertases, which amplify complement activation and opsonization of the bacteria. Clustering of C3b molecules around the surface-bound C3-convertase generates the C5 convertases that cleave C5 into C5a and C5b. C5b initiates the formation of the membrane attack complex that may lead to bacterial lysis, while C5a facilitates the recruitment of leukocytes to the site of the infection. C3b in the bacterial surface is inactivated to iC3b and C3dg. iC3b is a major ligand for the complement receptors CR3 and CR4 that mediate the phagocytosis of the microorganism by macrophages and other phagocytic leukocytes.

Complement activation is strictly controlled by regulatory proteins that maintain the homeostasis of the system and prevent damage to self-tissues. Factor H (FH), a 155-kDa circulating plasma glycoprotein that consists of 20 short consensus repeats (SCR) domains, is the master complement regulator (8). FH binds to C3b by its N- and C- terminal domains, both in the fluid phase and deposited on surfaces, and acts as a cofactor for Factor I to proteolyze C3b into inactive C3b (iC3b) or accelerates the decay of the C3 convertase (9). Not surprisingly, binding FH from the host plasma is a common strategy exploited by many bacterial pathogens to elude the complement system attack (10, 11).

In humans, the *CFH* gene is located in chromosome 1 adjacent to the genes encoding the complement FH-related proteins 1 to 5 (*CFHR-1-5*). Although the FHRs proteins have evolved from gene duplications of the *CFH* gene, they lack the FH regulatory domains and cannot regulate the complement system. In contrast, the FHRs have conserved the domains that FH uses to bind ligands in host cells and pathogens and therefore can compete with FH for binding to these ligands (11). Ligand competition between FH and the FHRs is referred to as “FH-deregulation” and has been postulated as a mechanism to interfere the complement evasion mechanisms mediated by the incorporation of FH to the bacterial surface (9). However, whilst the biological role of FH binding to *P. aeruginosa* has been well determined (12), the biological consequences of the interaction of the FHRs with the bacterial surface are still poorly understood.

In this study, we focused on FHR-3 because it shows the highest degree of amino acid identity to the microbial binding domain present in the SCR6 and 7 of FH among FHRs proteins (**Supplementary Figure 1**). FHR3 is a plasma protein, which is expressed in four glycosylated forms with molecular masses ranging from 45 to 56 kDa (8). Initial estimations indicated that plasma concentration of FHR-3 was 50-100 μg/ml (13, 14). However, more recent studies using specific FHR-3 monoclonal antibodies have demonstrated that the concentration of FHR-3 in human plasma is remarkably as low as 0.14-4 μg/ml (15, 16).

In this manuscript, we investigated the effect of FHR-3 on the complement activation induced by *P. aeruginosa* and whether this FH related protein influences host defense against *P. aeruginosa* infections.

## Materials and methods

### Bacterial strains

PAO1 and its isogenic *wzz2*-deficient mutant (PAO1Δ*wzz2*) were previously described (17). *wzz2* gene encodes a lipopolysaccharide (LPS) O antigen chain length regulator. Interruption of *wzz2* gene results in production of LPS devoid of the very long O antigen, conferring a serum-sensitive phenotype to the strain.

Two *P. aeruginosa* clinical strains, B205 and B75, collected from patients with bloodstream infection were also used in this study. Bacterial cells were grown in Luria Bertani (LB) broth at 37°C with shaking or in LB solidified with 1.5% agar.

### Human serum and complement proteins

Blood samples were collected from two donors with an FHR-1/FHR-3 deficiency. Human samples were taken after informed consent of each participant. The study was aligned with the Helsinki Declaration and was approved by the Institutional Review Board and the Regional Ethics Committee. Blood was coagulated at 37°C for 30 min and then centrifuged at 4,500 x g for 20 min at 4°C to separate the serum. Equal volumes of serum from the two donors with FHR-1/FHR-3 deficiency were mixed to get a pool of FHR-3-deficient human sera (ΔFHR-3). Human serum was aliquoted and stored at −80°C until its use. In some experiments, sera were heat inactivated (HI) at 56°C for 30 min prior to use.

Factor H and recombinant human FHR-3 proteins were supplied by HycultBiotech and Elabscience, respectively.

### Purification of EF-Tu

*P. aeruginosa* EF-Tu was purified from strain PAO1 harboring the plasmid pUCP18ApGw(tufB) which encodes His-tagged EF-Tu (18) using nickel-nitrilotriacetic acid (Ni-NTA) agarose (Qiagen, Valencia, CA) according to the manufacturer’s instructions.

Briefly, bacterial cells were suspended in lysis buffer consisting of 50 mM sodium phosphate (pH 8), 300 mM NaCl, 10 mM imidazole, and lysozyme (1 mg/ml) (Sigma- Aldrich, St. Louis, MO). The cells were disrupted by sonication (10 sets of 30-s pulses), and the resulting homogenate was centrifuged at 12,000 X *g* to remove cellular debris. Ni-NTA (5 ml in a 50% slurry) was added to the resulting supernatant, and the suspension was swirled at 4°C at 100 rpm for 1 h on a rotary shaker. The Ni-NTA resin was washed three times with 20 ml of wash buffer consisting of 50 mM sodium phosphate buffer (pH 8.0), 300 mM NaCl, and 20 mM imidazole. The His-tagged protein was eluted with 1.5 ml of 50 mM sodium phosphate buffer, pH 8.0, containing 300 mM NaCl and 250 mM imidazole. Purity (>97%) was confirmed by SDS-PAGE analysis.

### FH and FHR-3 binding assays

Binding of FH to bacterial cells was determined by whole-cell ELISA. Briefly, 96-well round-bottom polystyrene microtiter plates were coated overnight at 37°C with 1x10^8^ bacterial cells resuspended in phosphate-buffered saline (PBS). Next, wells were blocked with PBS containing 1% bovine serum albumin (PBS-BSA) and incubated with PBS as negative control, FH (20 µg/ml), recombinant human FHR-3 protein at different concentrations depending on the experiment or FHR-3-deficient human sera (10%) diluted in PBS. FH was detected with the specific mouse mAb antibody OX-24 (Abcam). Finally, wells were incubated with an alkaline phosphatase-conjugated goat anti-mouse immunoglobulin G (Sigma), and developed with p-nitrophenyl phosphate (Sigma) in 50mM carbonate-bicarbonate buffer, pH 9.6, 5mM MgCl_2_. Absorbance was measured at 415 nm. Negative controls values were < 0.1 optical density units and were subtracted from the experimental values. Washing steps with PBS were included between incubations that were performed for 1 h at 37°C.

Binding of FHR-3 to EF-Tu was also determined by ELISA using the protocol described above using microtiter plate wells that were coated overnight at 4°C with 1 µg of purified EF-Tu dissolved in 100 µl of PBS. FHR-3 was detected with the mouse mAb antibody MBI-6 (19).

To identify the binding region of FHR-3 and FH in EF-Tu, we generated a library of 25 synthetic N-terminal biotinylated 20-mer overlapping peptides spanning the entire *P. aeruginosa* EF-Tu molecule. Peptides dissolved in PBS-Tween 0.05% at 10 µg/ml were captured on streptavidin-coated microtiter plates overnight at 25°C and incubated for 1 h at 37°C with recombinant human FHR-3 (2 µg/ml) or purified human FH (10 µg/ml) diluted in PBS. After three washing steps, FHR-3 and FH were detected using the specific mouse mAb antibodies MBI-6 or OX-24, respectively. Primary antibodies were detected as described above.

### C3 deposition assays

Deposition of C3 on the bacterial cells was also determined by whole-cell ELISA. Microtiter plates were coated and blocked as described above and incubated for 15 min at 37°C with FHR-3-deficient human sera (25%) diluted in PBS supplemented with FHR-3 (2 µg/ml or 100 µg/ml of serum) or not. HI-serum was used as control. Next, microtiter plate wells were incubated sequentially with a mouse mAb anti-human C3 that recognizes an epitope in the ß-chain (C3-12.17) (20), an alkaline phosphatase-labeled goat anti-mouse immunoglobulin G (Sigma), and developed with p-nitrophenyl phosphate (Sigma) in 50 mM carbonate-bicarbonate buffer (pH 9.6) plus 5 mM MgCl_2_.

### C3a and C5a quantification

Bacterial cells (1x10^9^ CFU) of *P. aeruginosa* were incubated for 15 min at 37°C in FHR-3-deficient human sera (25%) diluted in PBS supplemented with FHR-3 (500 ng/ml or at 25 µg/ml) or not. FHR-3-deficient human sera incubated with FHR-3 without bacteria was used as negative control. After incubation, bacterial suspension was centrifuged and the amount of C3a and C5a present in the supernatant was quantified using the Human C3a/Complement C3a ELISA (Invitrogen) or the Human C5a/Complement C5a ELISA Kit (Invitrogen) following the manufacturer’s instructions.

### Serum resistance assays

Serum resistance assays were performed as previously described (21, 22). Briefly, a total of 1x10^6^ CFU of *P. aeruginosa* grown exponentially at 37°C in LB were incubated at 37°C in FHR-3-deficient human sera diluted in PBS supplemented or not with recombinant FHR-3. Optimal serum concentrations were determined empirically for each strain in the current setting. HI- FHR-3-deficient human sera was used as control. At different time points, survival was determined by counting colonies on LB agar plates.

### Statistical analysis

Comparisons among experimental groups were assessed using one-way ANOVA with multiple comparisons. All results are reported as the mean with standard deviation. Differences were considered statistically significant at *P* < 0.05.

## Results

### FHR-3 competes with FH for the binding to *P. aeruginosa*

**Figure 1:**
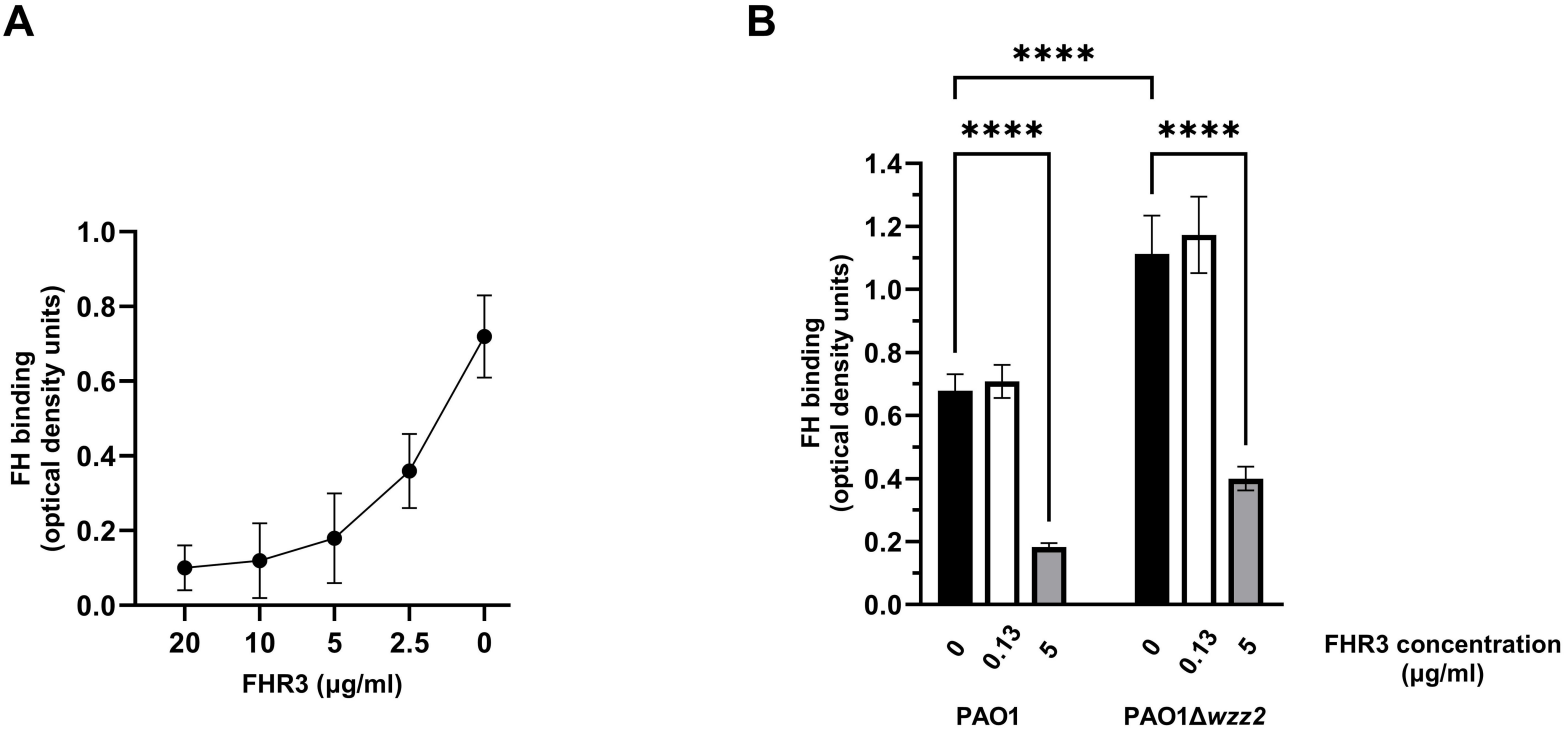
FHR-3 competes with FH for the binding to *P. aeruginosa.* ELISA binding assays of purified FH to the reference strain PAO1 and the serum-sensitive isogenic LPS-deficient strain PAO1Δ*wzz2* in the presence of FHR-3. In **(A)** the strain PAO1 was incubated with purified FH (20 µg/ml) in the presence of decreasing amounts of recombinant FHR-3 (x-axis on a logarithmic scale). In **(B)** the strains were incubated with purified FH (20 µg/ml) in the absence (black columns) or presence of FHR-3 at 0.13 µg/ml (white columns) or at 5 µg/ml (grey columns). Bound FH was detected with the mAb OX-24. Bars represents the mean of at least three independent experiments done in duplicate, and SD is indicated by error bars. Statistical analyses were performed using one-way ANOVA with multiple comparisons; *P* values are indicated on the bars with asterisks. ****P < 0.0001.

The competition for ligands between FH and the FHRs is termed “FH-deregulation” and has been proposed as a mechanism to disrupt the complement evasion strategies facilitated by the binding of FH to the bacterial surface (9). We evaluated the impact of FHR-3 on the binding of FH to *P. aeruginosa* using purified proteins. Bacterial cells were incubated with FH, and decreasing amounts of recombinant FHR-3, and the binding of FH to the bacterial surface was determined using a FH specific monoclonal antibody. FHR-3 reduced the amount of FH bound to *P. aeruginosa* PAO1 in a dose-dependent manner (**Figure 1A**). When the amount of recombinant FHR-3 and FH were at approximately equimolar ratios to those determined in normal human serum by Pouw et al. (15, 16) (FH: FHR-3 of 1:0.0066), FHR-3 had no effect on the binding of FH to the bacteria (**Figure 1B**). By contrast, at FH: FHR-3 ratios to those estimated in normal human serum by Fritsche et al. (13) (FH: FHR-3 of 1:0.27), FH bound on the bacterial surface of PAO1 was approximately 30% that seen in the absence of FHR-3 (**Figure 1B**). A similar pattern was observed when we determined the impact of FHR-3 on the binding of FH to the serum-sensitive strain PAO1Δ*wzz2* (**Figure 1B**). These results demonstrate antagonism between FH and FHR-3 for binding to the bacterial surface and suggest that FH and FHR-3 share a common binding site on *P. aeruginosa.*

**Figure 2:**
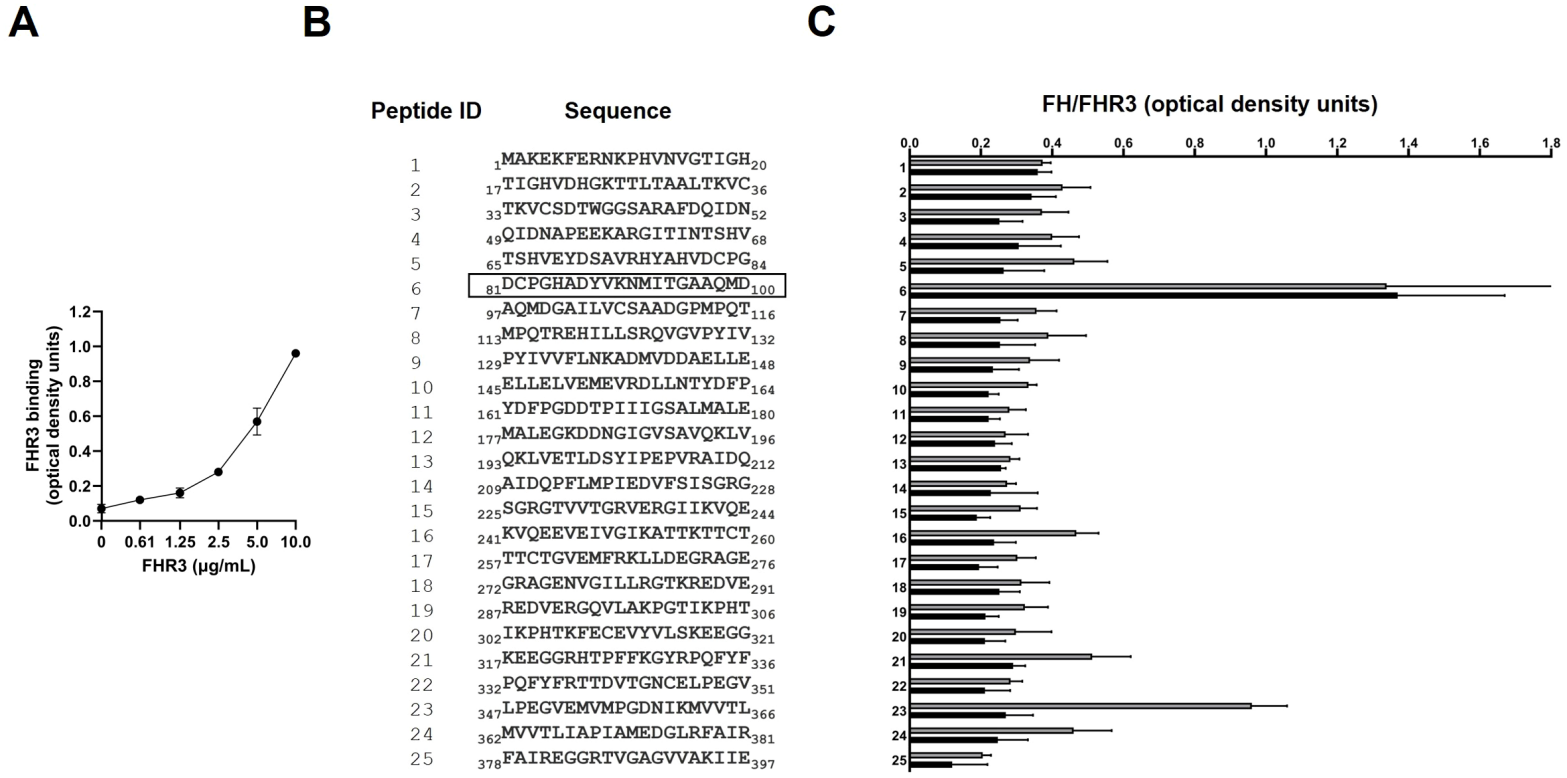
FHR-3 binds *P. aeruginosa* EF-Tu at the same site as FH. **(A)** The binding of recombinant FHR-3 to immobilized EF-Tu was analyzed by ELISA. Bound FHR-3 was detected with a specific monoclonal antibody. Data represent three experiments done in duplicate, and SD is indicated by error bars. **(B)** The sequence of the synthetic peptides (Peptide ID) covering the EF-Tu protein sequence that were used to identify FHR-3 and FH binding regions are shown. The black outline shows the Peptide ID that reacted with FHR-3 and FH. **(C)** The reactivity of recombinant FHR-3 (black columns) or FH (grey columns) was tested against 25 synthetic biotinylated EF-Tu 20-mer overlapping peptides by ELISA. FHR-3 and FH were detected with a specific mAb, respectively. Data represent three experiments done in duplicate, and SD is indicated by error bars shown.

It is known that EF-Tu is a surface-exposed protein that mediates the binding of FH to *P. aeruginosa* (12). FH has two binding domains for EF-Tu; one is located in the SCRs 6-7 and the other in the SCRs 18-20 (12). Given the high homology between FH SCRs 6-7 and FHR-3 SCRs 1-2 (**Supplementary Figure 1**), we hypothesized that EF-Tu might be an FHR-3-binding protein of *P. aeruginosa*. To test this hypothesis, we purified recombinant *P. aeruginosa* EF-Tu and conducted FHR-3 binding experiments. ELISA assays of recombinant EF-Tu incubated with recombinant human FHR-3 demonstrated that EF-Tu binds FHR-3 (**Figure 2A**). Furthermore, this binding was dose-dependent, as shown for the different concentrations of FHR-3.

To localize the binding region of FHR-3 and FH in EF-Tu, we used a library of synthetic N-terminal biotinylated peptides spanning the entire *P. aeruginosa* EF-Tu molecule (**Figure 2B**). Peptide mapping was performed using recombinant FHR-3, or purified human FH. Quantitative ELISA analysis revealed that the highest reactivity of both proteins was against peptide ID 6 (**Figure 2C**). FHR-3 reacted almost exclusively with peptide ID 6, while FH was also bound to peptide ID 23.

### FHR-3 increases the binding of FH to *P. aeruginosa* in human serum

**Figure 3:**
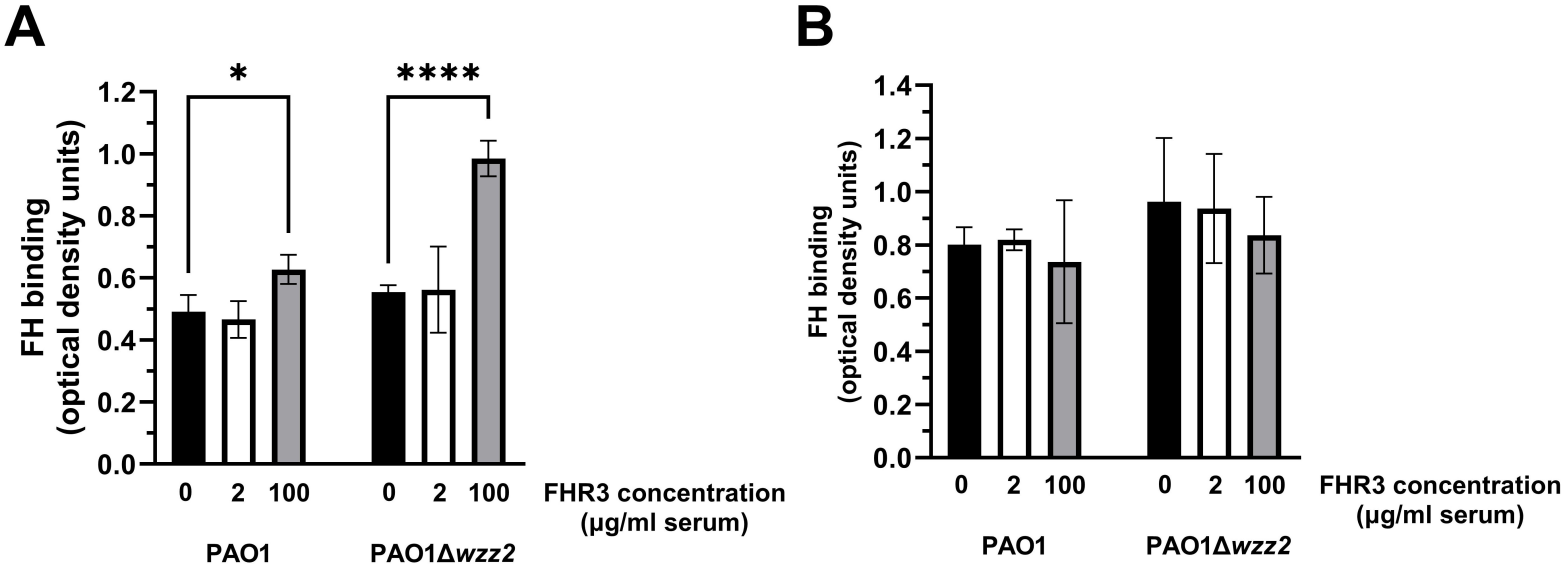
FHR-3 enhances the binding of FH to *P. aeruginosa* in human serum. ELISA binding assays of FH to the serum-resistant strain PAO1 or the isogenic LPS-deficient serum-sensitive strain PAO1Δ*wzz2* incubated in FHR-3-deficient serum **(A)** or heat-inactivated FHR-3-deficient serum **(B)** (both at 10%) without (black columns) or supplemented with FHR-3 at 2 µg/ml of serum (white columns) or 100 µg/ml of serum (grey columns). FH was detected with the mAb OX-24. Bars represents the mean of at least three independent experiments done in duplicate, and SD is indicated by error bars. Statistical analyses were performed using one-way ANOVA with multiple comparisons; *P* values are indicated on the bars. * P< 0.05, **** P <0.001.

We next assessed the influence of FHR-3 on the binding of FH to *P. aeruginosa* using FHR-3-deficient human sera (ΔFHR-3) at 10% supplemented with FHR-3 at 100 μg/ml or 2 μg/ml of serum, according to Fritsche et al. (13) or Pouw et al. (15), respectively. Conversely to the results obtained with purified FH, *P. aeruginosa* incubated with human ΔFHR-3, supplemented with FHR-3 at 100 μg/ml of serum, acquired higher levels of FH than those without exogenous FHR-3 (**Figure 3A**). Furthermore, the effect of FHR-3 on FH binding to *P. aeruginosa* was more remarkable on the serum-sensitive strain PAO1Δ*wzz2* than on the serum-resistant strain PAO1, with increment rates of approximately 100% and 20%, respectively. FHR-3 at 2 μg/ml of serum did not affect the binding of FH to *P. aeruginosa.* Interestingly, FHR-3 had no impact on the binding of FH to the bacteria when human serum was heat-inactivated (HI-ΔFHR-3) (**Figure 3B**), suggesting that the influence of FHR-3 on the binding of FH to the bacteria requires a functional complement system and may be attributed to the formation of C3b, the natural ligand of both proteins. These results lead us to hypothesize that FHR-3 bound to C3b facilitates the assembly of C3 convertase on *P. aeruginosa*, resulting in a much higher deposition of C3b that recruits FH rather than competing with FH for the binding to C3b.

**Figure 4:**
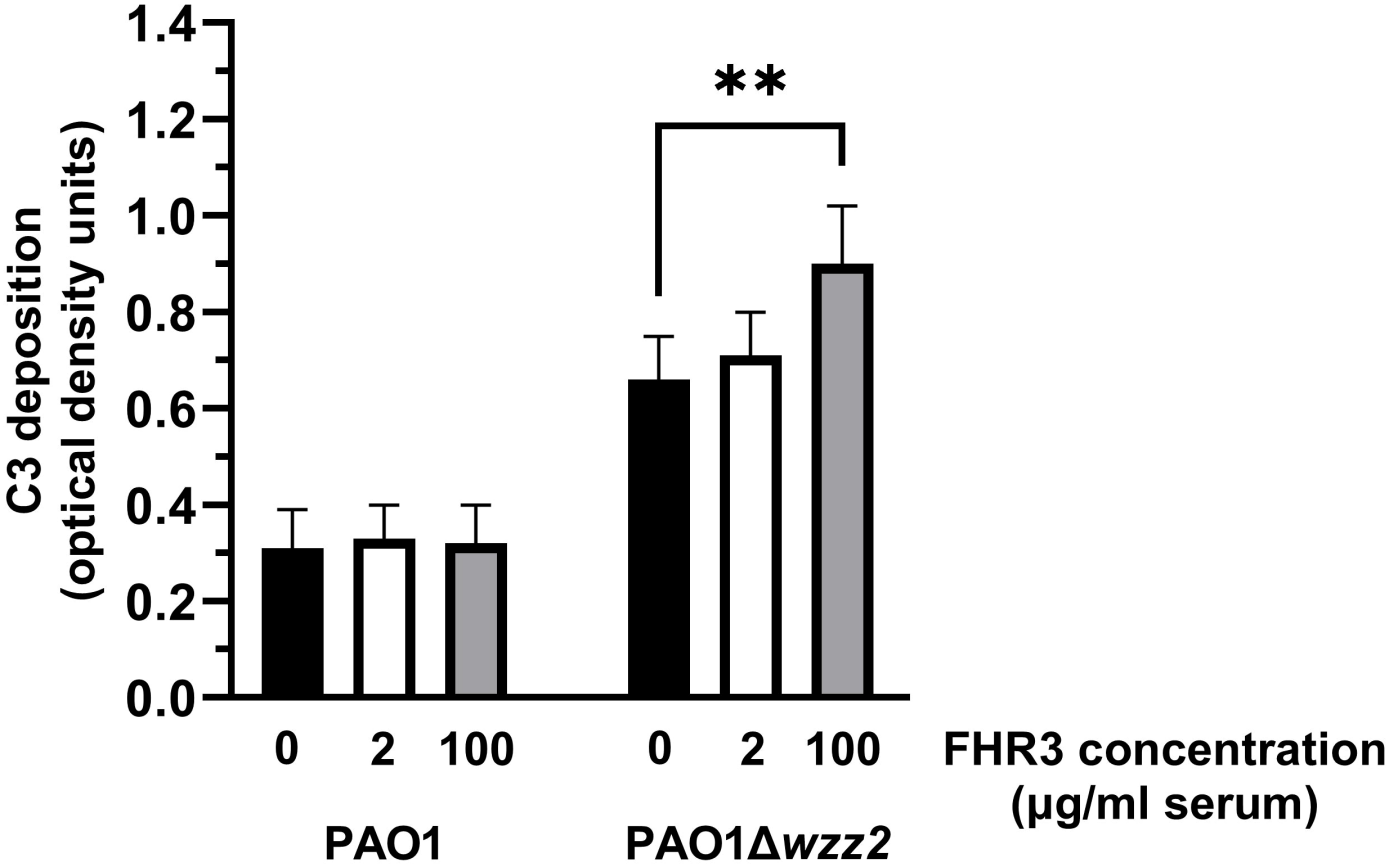
FHR-3 increases the deposition of C3 on *P. aeruginosa.* FHR-3 deficient serum (25%) without (black columns) or supplemented with FHR-3 at 2 µg/ml of serum (white columns) or at 100 µg/ml of serum (grey columns) were incubated for 15 min at 37°C with PAO1 or the isogenic serum-sensitive strain PAO1Δ*wzz2*. Deposition of C3 on the bacterial surface was determined by ELISA. Bars represents the mean of at least three independent experiments done in duplicate, and SD is indicated by error bars. Statistical analyses were performed using one-way ANOVA with multiple comparisons; *P* values are indicated on the bars. ** P< 0.01.

To test this hypothesis, we determined the effect of FHR-3 on the deposition of C3 on the bacteria (**Figure 4**). FHR-3 deficient serum (25%) supplemented with FHR-3 at 100 μg/ml of serum increased the amount of C3 fragments deposited on the serum-sensitive strain PAO1Δ*wzz2*, but had no effect on the serum-resistant strain PAO1 (**Figure 4**). Low concentrations of FHR-3 (2 μg/ml of serum) had no effect on the deposition of C3 fragments on *P. aeruginosa*.

**Figure 5:**
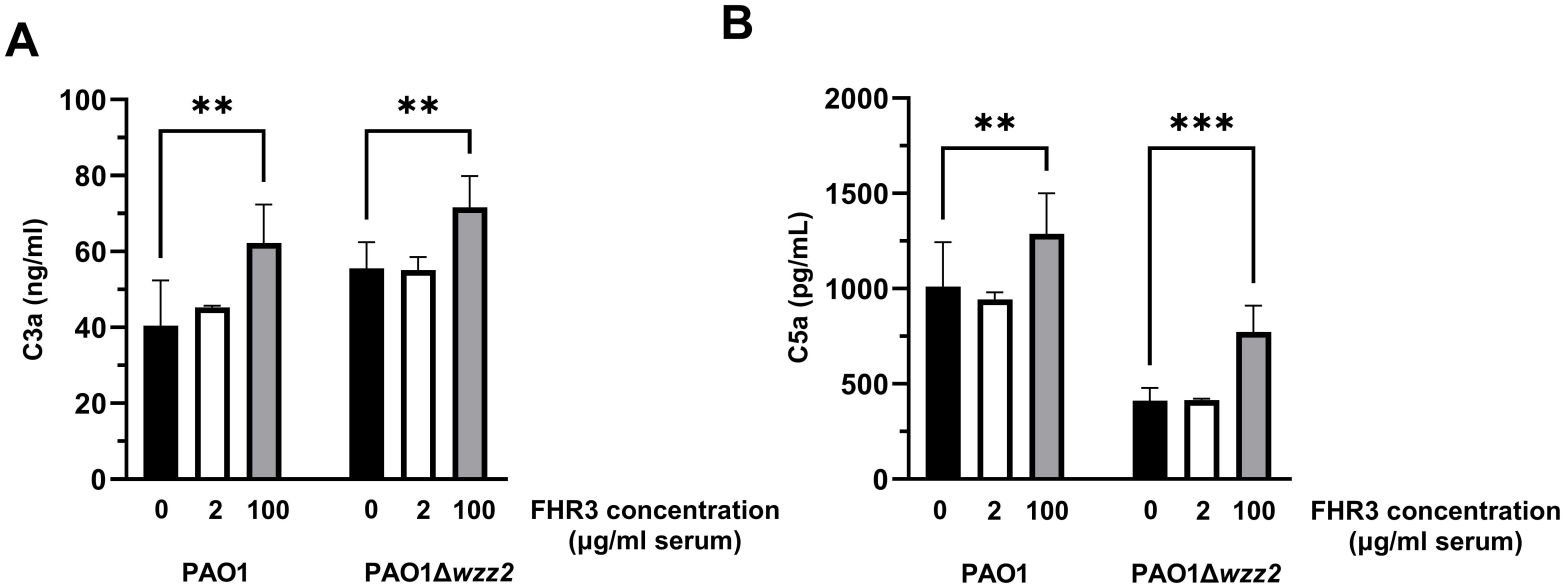
*P. aeruginosa*-induced generation of C3a and C5a is enhanced by FHR-3. FHR-3 deficient serum (25%) without (black columns) or supplemented with FHR-3 at 2 µg/ml of serum (white columns) or at 100 µg/ml of serum (grey columns) were incubated for 15 min at 37°C with the serum-resistant strain PAO1 or the isogenic serum-sensitive strain PAO1Δ*wzz2*. Levels of C3a **(A)** and C5a **(B)** were determined by ELISA. Bars represents the mean of at least three independent experiments done in duplicate, and SD is indicated by error bars. Statistical analyses were performed using one-way ANOVA with multiple comparisons; *P* values are indicated on the bars. ** P<0.01, *** P<0.001.

We also quantified the amount of generated C3a to directly assess the FHR-3-induced C3 convertase activity (**Figure 5A**). The addition of FHR-3 at 100 μg/ml of serum to the FHR-3 deficient serum increased the production of C3a induced by the *P. aeruginosa* independently of the strain tested. As anticipated, the serum lacking FHR-3 and supplemented with FHR-3 at 2 μg/ml of serum showed a production of C3a comparable to that observed without supplementation.

To further confirm that FHR-3 enhanced complement activation, we went one step further. We evaluated the formation of C5a in the human sera incubated with the bacteria in the presence or not of exogenous FHR-3 (**Figure 5B**). Consistent with the previous results, both strains produced higher levels of C5a when incubated in sera supplemented with FHR-3 at 100 μg/ml demonstrating that, at this concentration, this protein promotes the *P. aeruginosa-*induced activation of the complement system. However, low concentrations of FHR-3 (at 2 μg/ml of serum) had no effect on the production of C5a.

Overall, these results indicate that FHR-3, at the serum concentrations estimated by Fritsche et al. (13), enhances the activation and deposition of C3 on *P. aeruginosa*. In contrast, we did not observe any effect at the serum concentrations reported by Pouw et al. (15).

### FHR-3 promotes the killing of *P. aeruginosa*

**Figure 6:**
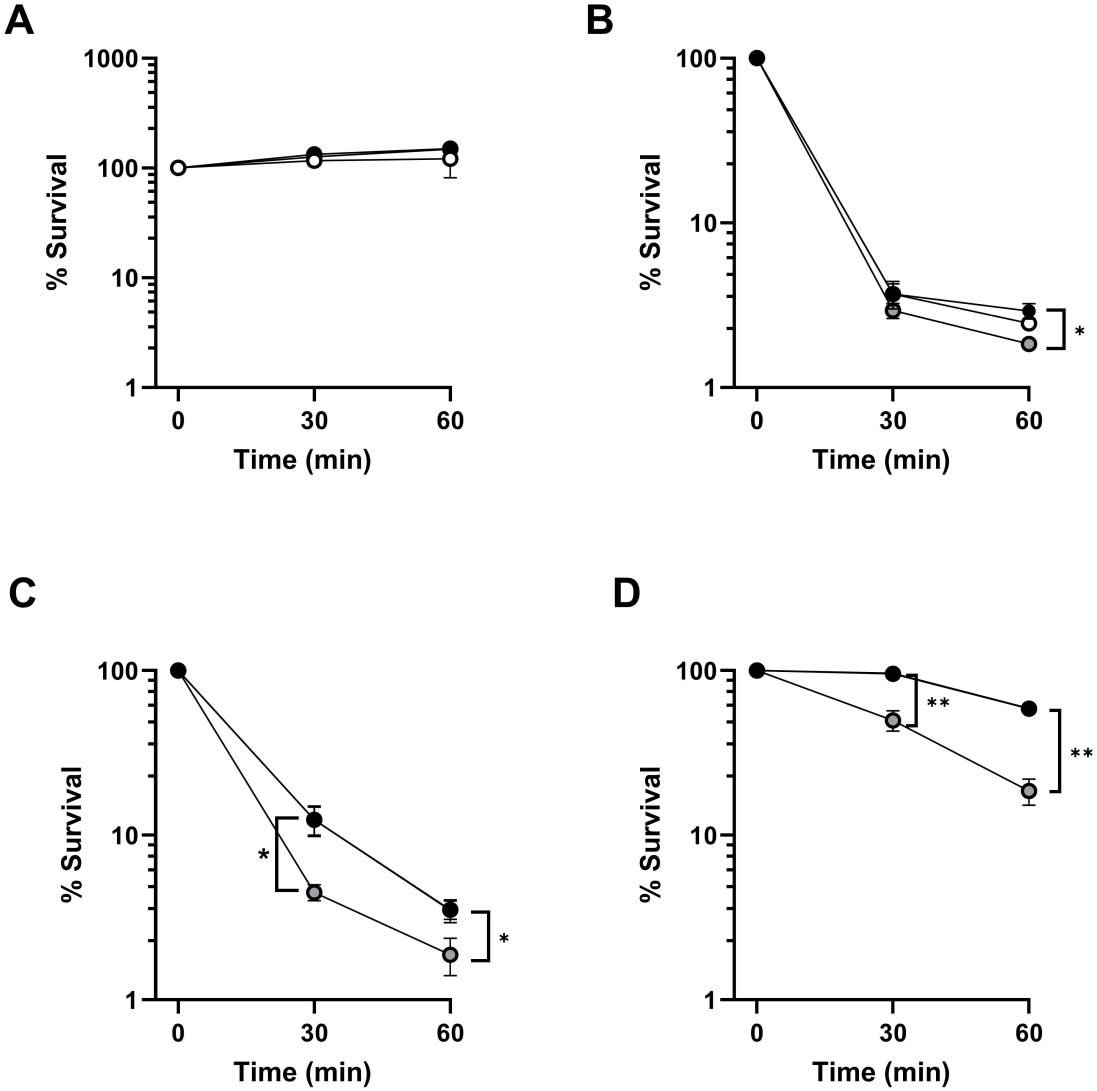
FHR-3 increases *P. aeruginosa* complement-mediated killing. Survival of strains PAO1 **(A)**, PAO1Δ*wzz2* **(B)**, and the clinical isolates from bloodstream infections B75 **(C)** and B205 **(D)** at different time points after incubation in strain-optimized concentrations of FHR-3 deficient serum (PAO1, 45%; PAO1Δ*wzz2*, 2.5%; B75, 2.5% and B205; 45%) not supplemented with FHR-3 (black circles) or supplemented with FHR-3 at 2 µg/ml of serum (white circles), or 100 µg/ml of serum (grey circles). Black and grey circles are overlaid in panel A, while black and white circles are overlaid in panels C and **(D)** Percentage survival at each time point was calculated in respect to the number of viable bacteria at time 0. Dots represent the mean of at least three independent experiments done in duplicate, and SD is indicated by error bars. Statistical analyses were performed using one-way ANOVA with multiple comparisons. *P* values; * P< 0.05, ** P <0.01.

Cleavage of C5 into C5a and C5b initiates the formation of the membrane attack complex that may result in the lysis of the microorganism. To investigate whether FHR-3-enhanced complement activation impacts on bacterial survival, we evaluated the viability of *P. aeruginosa* incubated in strain-optimized concentrations of ΔFHR-3 serum supplemented or not with recombinant FHR-3 (**Figure 6**). The addition of FHR-3 at any concentration did not impair the survival rate of the highly serum-resistant strain PAO1, which was able to grow in the human sera at 45%. On the other hand, the serum-sensitive strain PAO1Δ*wzz2* was efficiently cleared by the human sera (2.5%). Interestingly, the addition of FHR-3 at (100 μg/ml of serum) slightly increased the killing of this strain, but the addition of a low concentration of FHR-3 (2 μg/ml) did not enhance the clearance of this strain. This observation was further confirmed with two additional *P. aeruginosa* clinical strains isolated from bloodstream infections, B75 and B205. FHR-3 deficient human sera killed *P. aeruginosa* B75 and B205 more efficiently with the addition of FHR-3 at the concentration estimated by Fritsche et al. (13) (100 µg/ml of serum), than without it. The presence of FHR3 at the concentration reported by Pouw et al. (15) (2 µg/ml of serum) did not result in improved clearance of these isolates.

## Discussion

In this work, we have characterized a previously unrecognized interaction between the FHR-3, a member of the FH/FHRs protein family, and *P. aeruginosa*. It has been already reported that this microorganism binds different members of the FH protein family, including FH, FHL-1, and FHR-1 (12, 23, 24). *P. aeruginosa* utilizes FH and FHL-1 to inactivate C3b and to prevent formation of the C3 convertase to evade the host complement system (12, 23). However, the biological significance of the binding of the FHR proteins remained poorly investigated.

It has been suggested that FHRs proteins act as decoys molecules that displace FH from the surface of the pathogen due to their overlapping ligand spectrum with this complement inhibitor (9). Our competition studies using purified FH and FHR-3 support this idea. The most likely explanation for this result is that, given the high homology between FH SCRs 6-7 and FHR-3 SCRs 1-2 (**Supplementary Figure 1**), they share the same bacterial target. In this regard, it is noteworthy that most FH-binding microbial proteins also bind FHR proteins through those FH domains that are conserved among the FHR proteins (9, 25). *P. aeruginosa* is not an exception, therefore it is logical that the FH binding protein EF-Tu is also the ligand of FHR-3. A linear EF-Tu binding motif for both FH and FHR-3 was identified within domain 1 of this protein suggesting that this region of EF-Tu is surface exposed and thus accessible to ligand binding. In fact, comparisons of *P. aeruginosa* EF-Tu structure to EF-Tu structures from other microorganisms and three-dimensional modeling indicate that FH and FHR-3 binding region identified in this study is located on the surface exposed portion of the protein in the domain 1 distant from the typical consensus sequence of the GTP/GDP binding domain (26). Since EF-Tu is a well-conserved protein which is surface exposed in many different microorganisms, it is reasonable to hypothesize that this region may be a common FH microbial binding motif. Thus, the identification of the FH binding domain on EF-Tu may facilitate the generation of protective monoclonal antibodies to block the binding of FH to the pathogens avoiding complement inhibition.

When the competition experiments were performed using active human FHR-3-deficient sera supplemented with FHR-3, the results obtained were the opposite to those obtained with purified components. FHR-3 enhanced the binding of FH to the bacteria by a mechanism that is dependent of the complement activation, because in the experiments conducted with heat-inactivated serum, FHR-3 had no influence on the binding of FH to the bacteria. To explain this apparent contradiction, we postulate that surface-bound FHR-3 activates complement through the alternative pathway by promoting the formation of an active C3bBb convertase and the enhanced binding of FH to the bacteria was a consequence of the increased amount of C3b deposited on its surface, the natural ligand of FH.

The results of our experiments measuring the FHR-3-induced deposition of C3 and generation of C3a and C5a mediated by activation of the complement on *P. aeruginosa* supports this idea.

Our observations are in line with several lines of evidence supporting that the FHRs proteins promotes complement activation independently of FH (27–30). Further experiments will be required to characterize the underlying mechanisms that govern FHR-3-induced activation of the complement alternative pathway.

To our knowledge, only one study has provided direct evidence of the role of FHR-3 in bacterial infection. Caesar et al. demonstrated that FHR-3 competes with FH for binding to *Neisseria meningitidis* factor H binding protein (fHbp), promoting complement activation (31). Interestingly, these observations may explain the results of a genetic association study that linked a variant in the *CFHR-3* gene, associated with increased expression of the FHR-3 protein, with protection from *N. meningitidis* infection (32).

In our study, we show that FHR-3 enhances complement activation in the bacterial surface, which will result in increased amount of C3 deposition and killing of the bacteria. We believe that this effect of bacterial-bound FHR-3 increasing complement activation and opsonization may be a general strategy used by the innate immune system against different pathogens.

Our functional studies indicate that FHR-3 promotes the complement-mediated killing of *P. aeruginosa.* The effect of FHR-3 on the clearance of two bloodstream infection isolates was clear. However, FHR-3 had no impact on the viability of the highly serum-resistant strain PAO1 (7) or a poor effect on its isogenic highly susceptible mutant PAO1*Δwzz2*. This observation leads us to hypothesize that FHR-3 fine-tunes complement activation. Thus, its effect on bacterial clearance may rely on the specific phenotype of each isolate and the complement concentration at the site of infection. This observation is consistent with the high variable survival rates for different *P. aeruginosa* strains in blood (7).

It is noteworthy that the effect of FHR-3 on complement deposition and the killing of *P. aeruginosa* is only observed when we use levels of FHR-3 similar to those employed by Caesar et al. in their experiments with *N. meningitidis* (31). These levels are significantly higher than the physiological concentration of FHR-3 in human serum as subsequently reported by Pouw et al. (15). Indeed, in our experiments using FHR-3 concentrations within the range reported by Pouw et al., FHR-3 had no effect on complement activation on *P. aeruginosa*.

In light of these results, it is unlikely that FHR-3 levels in the blood significantly influence the outcome of *P. aeruginosa* infections. Nonetheless, our data does not rule out the possibility that the demonstrated effect of FHR-3 on complement deposition and killing of *P. aeruginosa in vitro* could occur *in vivo* in some infected tissues. Although a preliminary study suggests that FHR-3 does not function as an early major acute phase protein (15), other research has demonstrated that FHR-3 is more abundant in certain specific tissues when they are infected than in serum (33). Thus, *in vitro* studies are a limitation to investigate the effect of FHR-3 on bacterial infections and future studies should focus on the use of *P. aeruginosa Cfhr-3* knock-out mice model of infection to evaluate the role of this complement regulator factor *in vivo*.

Interestingly, *CFHR3* copy numbers impact on the FHR-3 serum levels and usually differ between individuals (15). Moreover, the deletion of *CFHR3* is a frequent polymorphism in humans that originated long time ago by a non-homologous recombination event. Its allelic frequency ranges from 0.5% in sub-Saharan populations to almost non-existence in Asiatic. In European populations it is around 2%, which translates into 4% homozygotes and 32% heterozygotes for the *CFHR3* deletion (15, 34). Future studies should be focused to know whether *CFHR3* deletion or low copy numbers of this gene is associated with an increased risk for the development of *P. aeruginosa* infections.

## Data Availability

The raw data supporting the conclusions of this article will be made available by the authors, without undue reservation.

## Supplementary material

Supplementary Figure 1 Schematic representation of the human FH, FHL-1 and FHR-3 Functional sites in FH are indicated in brackets on FH. Vertical alignment and colors show Short Consensus Repeats (SCR) homologous to SCRs 6-8 and 19-20 of FH. The degree of amino acid sequence identity (in percentage) between FH and FHR-3 is indicated below FHR-3 SCRs. FH-like protein 1 (FHL-1) is a splice variant of FH.
