# Supplementary figures and images for: Role of factor H-related protein 3 in Pseudomonas aeruginosa bloodstream infections
Source: Front Immunol. 2024 Sep 4;15:1449003. doi: 10.3389/fimmu.2024.1449003 (PMC11408224; doi:10.3389/fimmu.2024.1449003)

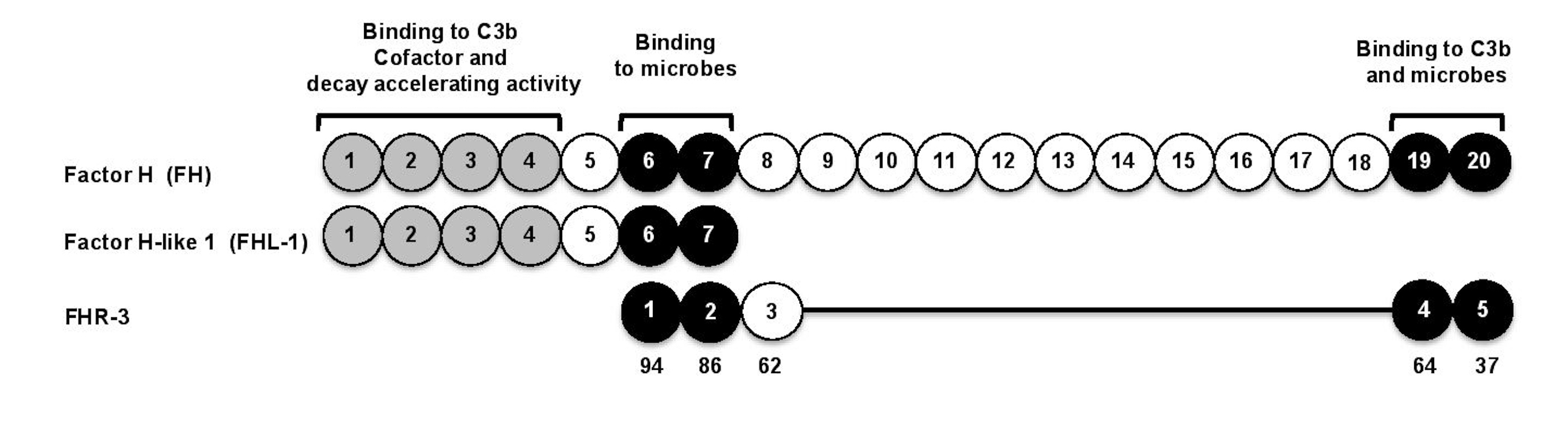

Supplement: Supplementary Figure 1 — Schematic representation of the human FH, FHL-1 and FHR-3 Functional sites in FH are indicated in brackets on FH. Vertical alignment and colors show Short Consensus Repeats (SCR) homologous to SCRs 6-8 and 19-20 of FH. The degree of amino acid sequence identity (in percentage) between FH and FHR-3 is indicated below FHR-3 SCRs. FH-like protein 1 (FHL-1) is a splice variant of FH. [file Image1.jpeg]
